# Advances in fecal microbiota transplantation for the treatment of diabetes mellitus

**DOI:** 10.3389/fcimb.2024.1370999

**Published:** 2024-04-10

**Authors:** Juan Zhang, Honggang Wang, Ying Liu, Min Shi, Minna Zhang, Hong Zhang, Juan Chen

**Affiliations:** ^1^ Department of Endocrinology, the Affiliated Huai’an No.1 People’s Hospital of Nanjing Medical University, Huai’an, Jiangsu, China; ^2^ Department of Gastroenterology, the Affiliated Huai’an No.1 People’s Hospital of Nanjing Medical University, Huai’an, Jiangsu, China

**Keywords:** fecal microbiota transplantation, diabetes mellitus, diabetes complications, gastrointestinal microbiome, metabolite, metabolic disease

## Abstract

Diabetes mellitus (DM) refers to a group of chronic diseases with global prevalence, characterized by persistent hyperglycemia resulting from various etiologies. DM can harm various organ systems and lead to acute or chronic complications, which severely endanger human well-being. Traditional treatment mainly involves controlling blood sugar levels through replacement therapy with drugs and insulin; however, some patients still find a satisfactory curative effect difficult to achieve. Extensive research has demonstrated a close correlation between enteric dysbacteriosis and the pathogenesis of various types of DM, paving the way for novel therapeutic approaches targeting the gut microbiota to manage DM. Fecal microbiota transplantation (FMT), a method for re-establishing the intestinal microbiome balance, offers new possibilities for treating diabetes. This article provides a comprehensive review of the correlation between DM and the gut microbiota, as well as the current advancements in FMT treatment for DM, using FMT as an illustrative example. This study aims to offer novel perspectives and establish a theoretical foundation for the clinical diagnosis and management of DM.

## 1 Introduction

**Figure 1:**
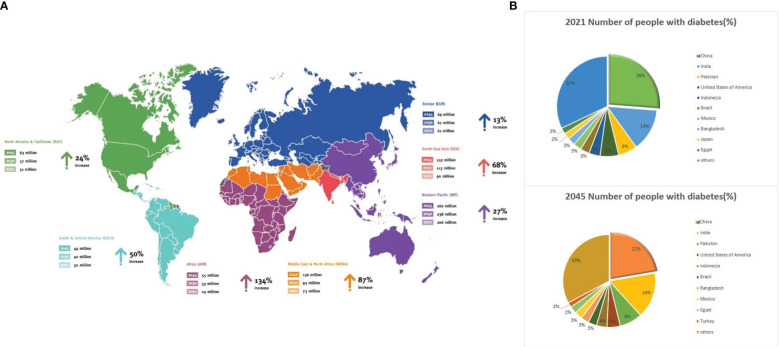
Global diabetes data. **(A)** Map 1: Number of people aged 20–79 years with diabetes worldwide and per IDF Region from 2021–2045. **(B)** Share of the global population with diabetes in 2021 and 2024. Source: https://www.diabetesatlas.org.

**Figure 2:**
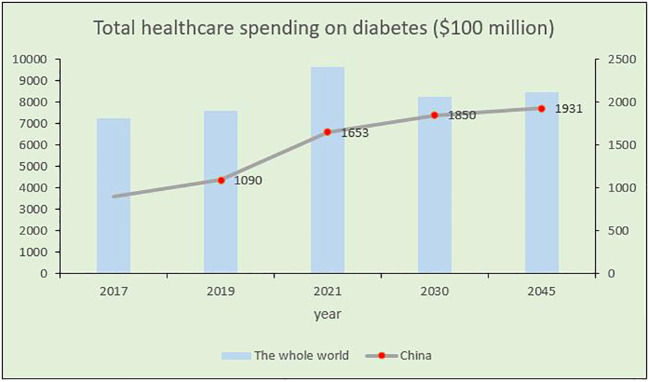
Trends of total medical expenditure on diabetes in China and worldwide in recent years.

DM is a metabolic disorder characterized by persistent high blood glucose levels resulting from the combined influence of genetic and environmental factors (Sapra and Bhandari, 2023). Prolonged hyperglycemia leads to chronic progressive lesions, functional decline, and failure in various organs and tissues, causing irreversible damage to multiple systems in the human body and threatening human health (Kolarić et al., 2022). Despite extensive research, DM, a typical chronic progressive disease, has no definitive cure. Current large public health and medical investments and a series of new diabetes drugs have not significantly improved DM control. According to the latest International Diabetes Federation (IDF) Diabetes Atlas, approximately 537 million adults (aged 20–79 years) worldwide are expected to develop DM by 2021. This number is expected to increase to 643 million by 2030 and 783 million by 2045 (Bommer et al., 2018; GBD 2021 Diabetes Collaborators, 2023; Zhong et al., 2023) (**Figure 1A**). The number of patients with diabetes in China will account for a quarter of the world’s diabetic population and may increase at a rate of 3%–5% every year (**Figure 1B**), thus imposing a serious social and economic burden (Bommer et al., 2018). Global diabetes-related healthcare spending is anticipated to reach $1.054 trillion by 2045, with complications and mortality accounting for approximately 5%–10% of the global economic burden of DM (**Figure 2**). Currently, the conventional treatment for DM involves controlling blood glucose levels with pharmacological and insulin replacement therapies (Ceriello et al., 2022; Fralick et al., 2022). However, these are only palliative and can result in adverse reactions and limitations. Furthermore, patient compliance, supply and quality of drugs, and time of diagnosis crucially impact the treatment of diabetes. Therefore, development of new and effective therapies to supplement existing clinical methods is essential.

The human microbiome comprises fungi, yeast, bacteria, archaea, and viruses. The intestine, which is one of the biggest organs in contact with the external environment after the skin, is home to a host of microorganisms, earning it the name of the “second human genome” (Gilbert et al., 2018; Aggarwal et al., 2023). However, evidence of symbiotic interactions between gut microbes and their hosts remains inconclusive. Homeostasis of the gut microbiota can benefit the host through immunomodulation, nutrient exchange, and metabolism of pathogenic microbes (Adak and Khan, 2019; Wang et al., 2023). Conversely, dysregulation of the gut microbiota can lead to various diseases. Numerous studies have indicated that the gut microbiota plays a crucial role in the development of various diseases through its metabolites and byproducts (Zhou et al., 2020; Fu et al., 2023b; Wang et al., 2023). The gut microbiota is the most abundant group of antigen-presenting cells (APCs) (Adak and Khan, 2019), and their imbalance disrupts immunity and homeostasis. By introducing environmental antigens into the gastrointestinal tract, the gut microbiota interferes with the reaction between antigens and the host immune system, resulting in increased intestinal permeability and the development of diabetes (Del Chierico et al., 2022; Guo et al., 2022; Zhou et al., 2022; Crudele et al., 2023; Wu et al., 2023).

The prevailing forms of diabetes include type 1 DM (T1DM), type 2 DM (T2DM), specific variations of diabetes, and gestational DM (GDM) (Sapra and Bhandari, 2023), which are categorized as chronic inflammatory diseases. Recent research has provided substantial evidence suggesting that the development of diabetes is influenced by environmental and genetic factors, as well as the gut microbiota. Microbiota crucially participate in mediating oxidative stress, insulin resistance, and chronic inflammation, thereby facilitating the progression of diabetes and its associated complications. Moreover, the gut microbiota of patients with diabetes show distinct degrees of disorder, and the abundance of *Firmicutes* and *Bacteroidetes* is particularly significant. Disorders in the gut microbiota can reduce insulin sensitivity and energy metabolism by altering the host intestinal mucosal barrier, thus affecting short-chain fatty acid (SCFA) synthesis, bile acid metabolism, and other pathways, ultimately leading to diabetes.

Therefore, re-establishing immune homeostasis by altering the form and number of the gut microbiota may be a potential intervention for diabetes. Fecal microbiota transplantation (FMT) is an intervention method that involves the transfer of the whole gut microbiota of a healthy donor into the intestine of a patient and has shown great clinical application owing to its safety, stability, convenience, and low incidence of side effects (Wang et al., 2022b; Porcari et al., 2023; Yang et al., 2023). Patients treated with FMT typically show reduced microbial diversity, abundance, and richness, compared with patients with normal gut microbiomes. In contrast, FMT can lead to the continued colonization of the intestine, thereby establishing a new microbiome. FMT has been extensively used in DM and its complications and is considered a promising treatment for DM when conventional hypoglycemic regimens remain ineffective. Consequently, a systematic review is needed to explore the mechanism of FMT in the treatment of diabetes and to improve its therapeutic effect.

## 2 Gut microbiota and DM

**Figure 3:**
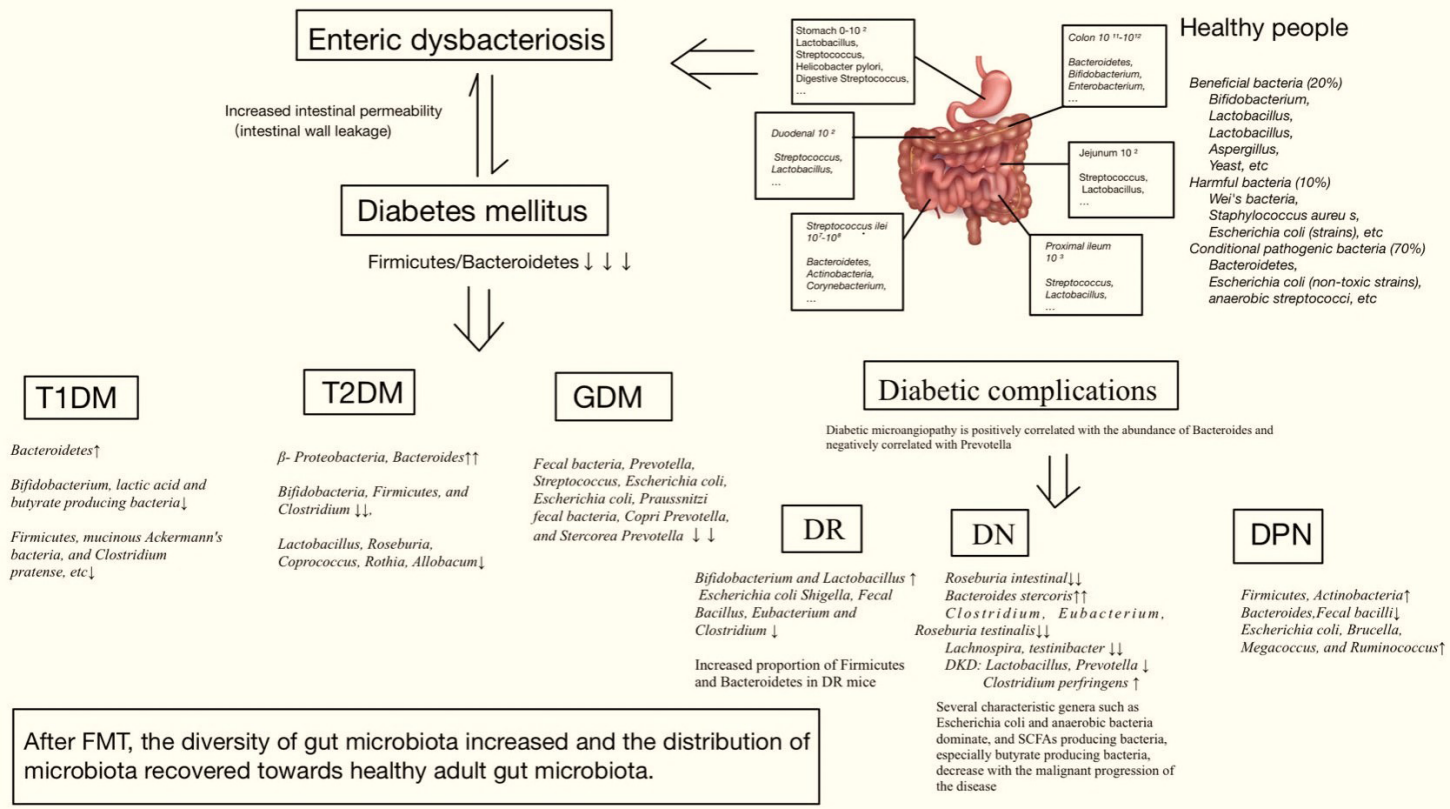
Relationship between gut microbiota and diabetes mellitus.

Through the analysis of human fecal samples collected using metagenomic sequencing (Schloissnig et al., 2013), dozens of phyla have been identified, including *Firmicutes*, *Bacteroidetes*, *Actinomyces*, *Proteobacteria*, *Fusobacteria*, *Micrococcus verrucosa*, *Cyanobacteria*, and *Spirochaeta* (de Vos et al., 2022). *Actinobacteria*, *Firmicutes*, *Proteobacteria*, and *Bacteroidetes* comprise 99% of the human bowel bacteria. The gut microbiota maintains host health and has been shown to be altered in various types of DM and its complications. Therefore, DM is associated with enteric dysbacteriosis, which damages the intestinal mucosal barrier and increases intestinal permeability. When the metabolite lipopolysaccharide (LPS) is translocated and released into the blood through a leaky intestinal mucosal barrier, a large amount of endotoxin is produced, which damages the function of islet β-cells, produces immune inflammation, activates macrophages, leads to vascular inflammation, and participates in the occurrence of DM (Zhang et al., 2021; Del Chierico et al., 2022; Guo et al., 2022; Lin et al., 2022; Zhou et al., 2022; Crudele et al., 2023; Wang et al., 2023; Wu et al., 2023) (**Figure 3**).

### 2.1 Gut microbiota and T1DM

T1DM is an autoimmune disease commonly observed in children and adolescents. It is mediated by T cells and characterized by the destruction of islet β-cells, absolute insulin deficiency, and hyperglycemia (Syed, 2022). Multiple studies have shown that, in contrast to the gut microbiota of healthy individuals, that of patients with T1DM is disordered in structure with decreased diversity (Diviccaro et al., 2023; Moreira et al., 2023), ultimately manifesting as an increase in the quantity of *Bacteroides* and a reduction in the quantity of *Bifidobacterium*, *lactic acid*, and *butyrate-producing bacteria* (Ma et al., 2020; Diviccaro et al., 2023; Moreira et al., 2023). Other studies (Demirci et al., 2020; Yuan et al., 2022) have found that, in contrast to those of non-diabetic groups, the proportions of *Ackermannia mucosus*, *Firmicutes*, and Clostridium praxobacter, as well as the proportions of *Firmicutes* and *Bacteroides* (F/B), were decreased in the T1DM group. After FMT, the numbers of *Bifidobacteria* and *Escherichia coli* increased, and with an increase in *Firmicutes* and a decrease in *Bacteroides* increasing the F/B ratio along with an increase in floral diversity, the distribution of gut microbiota approached normal (de Groot et al., 2021; He et al., 2022; Xie et al., 2022). Although T1DM is an autoimmune disease, patients may exhibit abnormal plasma metabolism (Zheng et al., 2022). In a longitudinal study, the early development of T1DM in infants was associated with reduced levels of sugar derivatives, amino acids, and fatty acids (including SCFAs), compared with controls (Huang et al., 2020). In addition, endogenous metabolites, including pyruvate, indoleacetic acid, and phenylacetylglycine, have been identified as potential biomarkers of T1DM (Wang et al., 2022).

### 2.2 Gut microbiota and T2DM

T2DM is an intricate polygenic disease with core pathological mechanisms involving islet dysfunction and insulin resistance (Mojsak et al., 2021; Sapra and Bhandari, 2023). Larsen et al. (2010) discovered that T2DM is associated with changes in the gut microbiota. Among them, *β-Proteobacteria* increased significantly, whereas *Bifidobacteria*, *Firmicutes*, and *Clostridium* decreased significantly, and the scale of *Bacteroides* and *Firmicutes*, as well as the proportion of *Prevobacteria* and *Clostridium globosum*, were positively correlated with blood glucose concentration. Existing research has indicated that *Bifidobacteria*, *Bacteroides*, *Fecalobacteria*, *Akkermansia muciniphila*, and *Rosanobacteria* are negatively correlated, whereas *Ruminococcus*, *Clostridium*, and *Cyanobacteria* are positively correlated with T2DM (Liu et al., 2022; Zhou et al., 2022; Liu et al., 2024; Pan et al., 2024). Studies suggest significant dissimilarities in the constitution and proportion of gut microbiota between patients with T2DM and those with normal glucose tolerance; overall, a reduced number of *Firmicutes* in participants with T2DM was observed (Fassatoui et al., 2019). In a controlled trial (Su et al., 2022), with the exacerbation of symptoms, the expression of *Bacteroides* in the gut microbiota of T2DM mice was high, whereas the expressions of *Lactobacillus*, *Roseburia*, *Coprococcus*, *Rothia*, and *Allobacum* decreased. In contrast, FMT-treated rats demonstrated a remarkable increase in this ratio. In addition, intestinal flora metabolites, such as bile acids, SCFAs, trimethylamine, tryptophan, and indole, to a certain extent, affected the reduced insulin sensitivity associated with T2DM dysfunction and regulated metabolism and immune homeostasis (Liu et al., 2022). Song et al. explored the relationship between the intestinal flora and differential metabolites in T2DM mice by identifying changes in urine metabolites (Song et al., 2023). Furthermore, 31 different metabolites, including phosphatidylcholine, that are identified by urine metabolomics may be closely related to the glycerophospholipid and arachidonic acid pathways. The results showed that the species diversity and abundance of intestinal flora in T2DM mice decreased, the levels of beneficial bacteria such as *lactobacillus* significantly decreased, and the levels of harmful bacteria such as *Helicobacter pylori* significantly increased. Common diabetes biomarkers, such as intravenous plasma glucose and hemoglobin A1c, are subject to individual limitations; therefore, identifying new specific metabolites can serve as important predictors of T2DM development. For example, serum indole propionic acid is negatively correlated with serum highly sensitive C-reactive protein levels, whereas indole propionic acid levels are lower in patients with impaired glucose tolerance, which is directly related to insulin secretion (Slouha et al., 2023). Therefore, in T2DM cynomolgus monkeys, four common serum lipids, phosphatidylcholine (18:0_22:4), lysophosphatidylcholine(14:0), phosphatidylethanolamine (PE) (16:1_18:2), and PE (18:0_22:4), were downregulated and identified as potential biomarkers, indicating that the glycerophospholipid pathway is related to its pathogenesis (Tian et al., 2024).

### 2.3 Gut microbiota and GDM

GDM refers to the progression of impaired glucose tolerance and diabetes during pregnancy (Kautzky-Willer et al., 2023). Previous studies (Koren et al., 2012; Sun et al., 2023) found that the abundances of *Proteobacteria*, *Actinobacteria*, and *Roseburia* in the intestines of healthy pregnant women changed throughout pregnancy. Throughout the gestational cycle, *Tyzzerella 4* and *Eisenbergiella* were enriched in the guts of patients with GDM during the first trimester. *Eisenbergiella* and *Taiseri*a showed a positive correlation with fasting blood glucose levels (Ma et al., 2020). During the third trimester in patients with GDM, high abundances of *Collinsella*, *Actinomycetes*, and *Rothia* were identified, and alterations in the gut microbiota persisted, even 8 months after childbirth (Crusell et al., 2018). Compared with the offspring of healthy pregnant women, patients with GDM had a lower alpha diversity of gut microbiota. The abundances of *Firmicutes*, *Bacteroidetes*, *Prevotella*, and *Lactobacillus* were also low (Su et al., 2018). Through comprehensive metagenomic and metabolomic analyses of a group of women with GDM and pregnant women with normal glucose tolerance, changes in the gut microbiome associated with both GDM and changes in circulating metabolites were identified (Ye et al., 2023). Compared with the control group, patients with GDM had significantly lower levels of SCFA-producing genera, including *Faecalis*, *Prevotella*, and *Streptococcus*, as well as *faecalis*, *Eubacterium Schlierii*, *prausnitzi*, *Prevotella copri*, and *Prevotella stercorea*. Additionally, 2-hydroxybutyric acid and L-alpha-aminobutyric acid were significantly increased, but methionine sulfoxide, allantoin, dopamine, and dopaminergic synapses were significantly decreased. These results suggest that insufficient dopamine circulation, unbalanced SCFA production, and excessive metabolic inflammation are important pathways for the gut microbiota to drive GDM development.

### 2.4 Gut microbiota and diabetic microvascular complications

Long-term elevated blood sugar can cause vascular disease, which can endanger the tissues and organs such as the eyes, heart, kidneys, brain, and nerves. According to World Health Organization statistics, up to 100 types of diabetic microvascular complications have occurred. Once diabetic complications occur, reverse treatment becomes difficult. The prevalent clinical complications of diabetic microvascular diseases include diabetic retinopathy (DR), diabetic peripheral vascular disease, diabetic nephropathy (DN), and diabetic neuropathy (Li et al., 2023; Sapra and Bhandari, 2023; Jia et al., 2024; Kanbour et al., 2024). Diabetic microangiopathy negatively correlates with *Prevotella* abundance and positively correlates with *Bacteroides* abundance, with changes in floral abundance, diversity, and proportion. The abundances of *Lactobacillus* and *Prevotella* decreased in patients with diabetic kidney disease, whereas the abundance of *Fusobacterium prevotella* increased. The F/B ratio was increased in DR mice. Metabolites such as LPS and SCFA affect neuronal homeostasis and participate in diabetic peripheral neuropathy (DPN). In addition, dysregulation of the metabolic pathways of gut-related products, such as tryptophan, vitamin B6, and purines, may be involved in the mechanism of diabetic complications associated with glucose homeostasis. In addition to sharing the metabolic pathways of DM, researchers have found that diabetic complications are associated with specific metabolite levels (Ma et al., 2024). Therefore, the microbiota influences variations in intestinal microecology in DM microangiopathy and is involved in the progression and prognosis of disease (Hong et al., 2022).

#### 2.4.1 Diabetic retinopathy

Diabetic retinopathy (DR) is a major cause of visual impairment and blindness in patients with advanced T2DM (Shukla and Tripathy, 2023; Gupta and Thool, 2024). It is a microvascular complication of DM associated with oxidative stress, activation of inflammatory response pathways, microvascular injury, and dysfunction. DR is also related to the abnormal regeneration of small blood vessels and changes in the structure and function of glial components (Madonna et al., 2017). The use of 16S rRNA gene sequencing to analyze the stool samples of patients with DM, patients with DR, and healthy controls (HC group) without retinopathy revealed differences in microbial structures and compositions among the three groups. Compared with that of the HC group, the diversity of α and β in the DR and DM groups was decreased, and the amounts of *Lactobacillus* and *Bifidobacterium* were increased. Notably, *Pasteurelliaceae* was reduced in the DR group, whereas it was increased in the DM group, suggesting that *Pasteurelliaceae bacteria* can be used as a microbial feature to distinguish between DR and DM (Huang et al., 2021; Hasani et al., 2024).

#### 2.4.2 Diabetic nephropathy

Diabetic nephropathy (DN) is a serious complication of diabetes and an important cause of death in patients (Varghese and Jialal, 2023; Das et al., 2024). Its clinical manifestations include proteinuria, hypertension, and edema. Studies have found that gut microbiota disorders are predisposing factors for DN. Patients with DN are characterized by an overgrowth of intestinal bacteria, accumulation of toxic compounds connected with the flora, disruption of intestinal barrier function, and chronic inflammatory response (Chi et al., 2021). The stool analysis results of 16S rRNA gene sequencing in patients with DN and healthy adults indicated that the multiplicity of patients’ flora was decreased. *Escherichia-Shigella* and *anaerobic bacteria* were dominant, whereas SCFA-producing bacteria, especially butyrate-producing bacteria, decreased with the malignant progression of the disease (Du et al., 2021). Using the meta-genomic sequencing of stool samples from an HC group and patients with T2DM with or without DN, Zhang et al (Zhang et al., 2022). identified gut microbiota imbalances in the stool samples from patients with DN, with the abundance of *Roseburia* consequentially decreasing and that of *Bacteroides stercoris* significantly increasing. Although the gut microbiota in these groups did not show any obvious distinction, the relative abundance of potential probiotics was visibly reduced in patients with diabetes. Renal function tests in DN often reveal decreased urinary albumin levels and glomerular filtration rates. Li et al. (2020) compared the consistency of the gut microbiota in mice with different outcomes during DN induction and found that *aerobic* and *anaerobic Bacillus* may exacerbate renal function deterioration and that *Blautia* may have a protective effect against DN. Therefore, the gut microbiota contributes to the regulation of renal function in this model.

#### 2.4.3 Diabetic peripheral neuropathy

Diabetic peripheral neuropathy (DPN) (Bodman and Varacallo, 2023; Zhu et al., 2024) is a type of diabetic neuropathy that mainly affects the distal lower extremities and is often characterized by sensory loss, numbness, pain, gait disturbances, and amputation. A study on the characteristics of the gut microbiota in DPN showed that the abundance of *Actinomycetes* and *Firmicutes* is increased in DPN, whereas that of *Bacteroides* is decreased. Specifically, the abundances of *Bacteroides* and *Faecalis* is decreased in DPN, whereas those of *Escherichia-Shigella*, *Braxella*, *Macrococcus*, and *Ruminococcus* are increased (Wang et al., 2020). The products of the gut microbiota are closely associated with nervous system homeostasis. Although relevant research is currently being conducted, the influence of FMT on DPN needs to be explored further.

### 2.5 Fecal microbiota transplantation

Chronic low-grade inflammation, abnormal SCFA levels, and impaired bile acid metabolism caused by enteric dysbacteriosis play important roles in the progression of metabolic diseases. Therefore, regulating and restoring homeostasis of the gut microbiota is a novel intestinal microecological therapy for treating diabetes (Okubo et al., 2018; Agus et al., 2021). While these methods include FMT (Zheng et al., 2022), prebiotics, probiotics, and antibiotics, FMT is the most direct and effective.

Approximately 3,000 years ago, India first used cow dung for the treatment of gastrointestinal diseases (Hoh and Dhanashree, 2017). In the Eastern Jin Dynasty (AD 317–420), Ge Hong recorded a therapeutic method similar to FMT, also known as “Yellow Long Tang,” for the treatment of food poisoning and diarrhea (Zheng et al., 2022). During World War II (Bárcena et al., 2019), German soldiers used camel feces to treat diarrhea. Currently, FMT is used to treat bacterial infections, and several clinical trials have validated FMT as a viable therapeutic option.

FMT generally delivers fecal microbiota from a thoroughly screened healthy donor into the small intestine via an oral capsule or duodenal tube, but it can also enter the large intestine via an enema or colonoscopy. Studies have compared the upper and lower digestive tract pathways of FMT and found no significant differences in cure rates between the two modes of administration (Youngster et al., 2014). However, the clinical resolution rate of the lower gastrointestinal pathway is higher than that of the upper gastrointestinal pathway in patients with *Clostridioides difficile* infection (CDI) (Quraishi et al., 2017). Oral bacterial liquid capsules and nasal feeding tubes have primarily been used (Green et al., 2020; Allegretti et al., 2021), and donor sources include autologous and allogeneic floral transplantations. In clinical studies on FMT in patients with diabetes (Aron-Wisnewsky et al., 2019; Belvoncikova et al., 2022; Hou et al., 2022; Zhang et al., 2022), a healthy human fecal donor who meets the relevant requirements is most commonly selected. When traditional hypoglycemic drugs are ineffective or cannot be tolerated, FMT, as a way to intervene in the gut microbiota, has a satisfactory overall effect with advantages such as high clinical safety and few adverse reactions (Wang et al., 2022a). **Table 1** displays the clinical studies related to the treatment of DM with FMT.

**Table 1:** Top 10 countries or territories for number of adults aged 20–79 years with diabetes in 2021 and 2045.

		2021			2045
Rank	Country or territory	Number of people with diabetes (millions)	Rank	Country or territory	Number of people with diabetes (millions)
1	China	140.9	1	China	174.4
2	India	74.2	2	India	124.9
3	Pakistan	33	3	Pakistan	62.2
4	United States of America	32.2	4	United States of America	36.3
5	Indonesia	19.5	5	Indonesia	28.6
6	Brazil	15.7	6	Brazil	23.2
7	Mexico	14.1	7	Bangladesh	22.3
8	Bangladesh	13.1	8	Mexico	21.2
9	Japan	11	9	Egypt	20
10	Egypt	10.9	10	Turkey	13.4
Total		537	Total		783

Source: https://www.diabetesatlas.org.

## 3 Role of gut microbiota in the pathophysiology of DM

**Figure 4:**
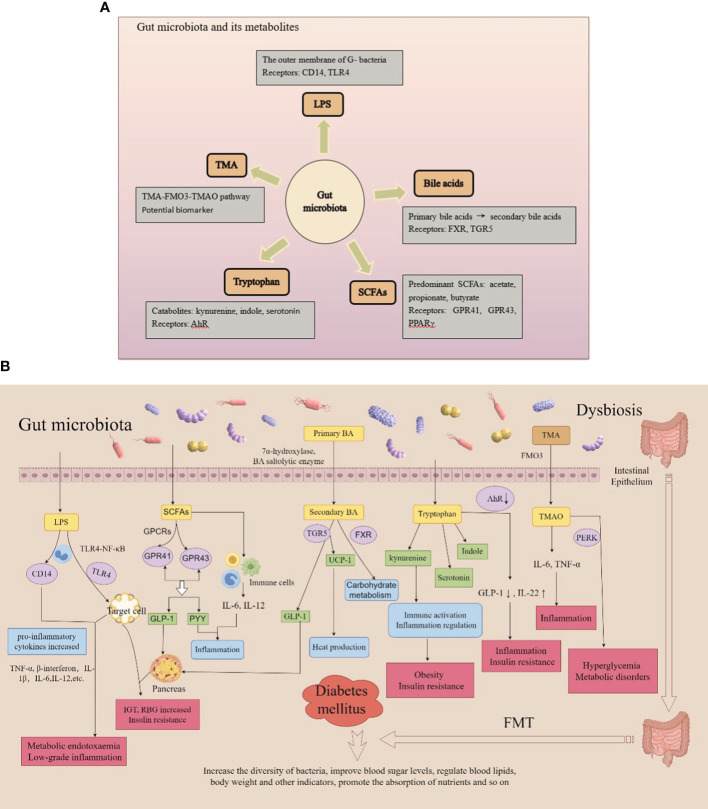
**(A)** Gut microbiota and its metabolites; **(B)** Metabolic mechanisms of the gut microbiota.

Recently, studies on various types of DM and its complications along with gut microbiota have been conducted (Yan et al., 2019; Arora et al., 2021; Ng et al., 2022). The gut microbiota of patients with diabetes differs from that of healthy counterparts. Specifically, the number of probiotic colonies, such as *Bifidobacterium* and *Firmicutes*, is decreased, and the number of pathogenic bacteria, such as *Enterococcus* and *Enterobacter*, is increased, resulting in an increase in inflammatory factors. In addition, these variations can induce the production of LPS, activate inflammatory responses, and affect the metabolism of SCFAs, bile acids, tryptophan, and trimethylamine N-oxide, thus promoting the development of DM (Fu et al., 2023a; Wu et al., 2023) (**Figures 4A, B**).

### 3.1 Gut microbiota: LPSs and DM

Endotoxins, also called LPSs, are a main component of the outer membrane of gram-negative bacteria and are involved in increasing pro-inflammatory cytokine levels and impairing pancreatic function. Variations in the gut microbiota in DM lead to various alterations, including an increase in the number of gram-negative bacteria, further release of LPS, and production of endotoxin, which damages the intestinal mucosa and results in leakage of the intestinal wall (Zhang et al., 2021; Christovich and Luo, 2022; Girdhar et al., 2022; Aleman et al., 2023) and endotoxemia (Lai et al., 2015; Velasquez, 2018). Subsequently, LPS can activate CD14 and toll-like receptor 4 (TLR4) on the surface of mononuclear macrophages, causing an increase in pro-inflammatory cytokines such as tumor necrosis factor α (TNF-α), β-interferon, and interleukin, and induce a series of chronic low-level inflammatory reactions in the body. The inflammation, in turn, interferes with insulin signaling, damaging islet β-cells and leading to absolute insufficiency of insulin secretion and insulin resistance (Siljander et al., 2019; Dedrick et al., 2020; Del Chierico et al., 2022; Girdhar et al., 2022; Luo et al., 2023). Cani et al. (2007) injected LPS subcutaneously into mice for 4 consecutive weeks to induce metabolic endotoxemia and chronic inflammation. After 4 weeks, the mice exhibited metabolic disorders characterized by impaired glucose tolerance, random blood glucose increases, and insulin resistance. Under the action of lipid polyglycoproteins, LPS binds to serum CD14 and promotes metabolic endotoxemia and low-grade inflammation (Zanoni and Granucci, 2013; Lee et al., 2019; Ciesielska et al., 2021; Mohammad and Thiemermann, 2021; Blanks et al., 2022). In addition, LPS activates the TLR4-nuclear factor-κB signal transduction pathway by combining with TLR4 on the surface of the cell membrane of host cells, thus causing target cells to secrete various inflammatory factors such as interleukin and TNF-α, which damage β-cells and reduce insulin secretion (Candelli et al., 2021; Ciesielska et al., 2021; Kim et al., 2022; Zhang et al., 2022; Li et al., 2023). Therefore, LPS-mediated chronic low-level inflammatory responses may be involved in the mechanisms underlying DM pathogenesis.

### 3.2 Gut microbiota: SCFAs and DM

SCFAs are a class of organic acids, including acetic acid, propionic acid, and butyric acid, generated by the fermentation of cellulose by the gut microbiota. The gut microbiota, especially *Bifidobacterium*, *Lactobacillus*, *Clostridium*, and *Bacteroidetes*, can produce abundant polysaccharide lyases and glycoside hydrolases that ferment carbohydrates into metabolic SCFAs. SCFAs regulate the gut microbiota, maintain fluid balance, inhibit the formation of intestinal inflammatory factors, and participate in intestinal immune responses and energy conversion. The G-protein-coupled receptors GPR41 and GPR43 are important regulatory factors that regulate glucose and lipid metabolism and can be directly activated by SCFAs to induce the formation of glucagon-like peptide 1 (GLP-1) and gastrointestinal polypeptide YY (PYY), which inhibit cellular inflammation and related immune diseases (Brar and Kohn, 2019; Roy et al., 2020; Zhang et al., 2023). Acetic acid can increase GLP-1 by binding to GPR43, thereby promoting insulin secretion. Propionic acid regulates gluconeogenesis via GPR41 and affects glucose metabolism. Butyrate stimulates the secretion of GLP-1 by activating peroxisome proliferator-activated receptor γ (PPARγ) and increases the number and activity of regulatory T-lymphocytes by inhibiting the recruitment of neutrophils and effector T-cells, ultimately reducing inflammation and enhance insulin sensitivity (He et al., 2020; Palmnäs-Bédard et al., 2022; Portincasa et al., 2022; Solar et al., 2023). Butyrate and acetate supplementation protects nonobese diabetic (NOD) mice from developing autoimmune diabetes (Mariño et al., 2017). Huang et al. (2020) compared the gut microbiota of healthy people and patients with T1DM and found that the gut microbiota of patients with diabetes could promote different IgA-mediated immune responses. SCFA treatment reduced the IgA response caused by intestinal bacteria as well as the severity of islet cell damage in NOD mice. SCFAs can stimulate intestinal L-cells to discharge the intestinal hormones GLP and PYY, thereby improving metabolic levels and increasing the absorption of energy substances (Wu et al., 2021). In contrast, SCFAs effectively enhance intestinal repair by repressing the activation of inflammatory cells and related responses (Salamone et al., 2021). Therefore, the gut microbiota is disturbed, and SCFA levels are significantly reduced, which can lead to diabetes.

### 3.3 Gut microbiota: Bile acids and DM

The gut microbiota and liver are involved in bile acid metabolism. Under the action of 7α-hydroxylase and bile acid saltolytic enzyme, primary bile acids transform into secondary bile acids while simultaneously activating a range of nuclear receptors, including the nuclear farnesoid X receptor (FXR) and G-protein-coupled bile acid receptor 1 (TGR5), thus facilitating both hepatic synthesis and intestinal reabsorption of bile acid. They additionally play significant roles in glucose, lipid, and energy metabolism (Liu et al., 2020; Zhao et al., 2020; Gao et al., 2022; Chen et al., 2023; Collins et al., 2023). Traussnigg et al. (2021) administered the non-steroidal FXR agonist PX-104 for four weeks in 12 non-diabetic patients with non-alcoholic fatty liver disease and found that the area under the C-peptide curve measured by oral glucose tolerance testing was significantly reduced, and 92% of the patients had improved insulin sensitivity. Therefore, FXR may be involved in glucose metabolism. Chen et al. discovered that the bile acid glycoursodeoxycholic acid (GUDCA) actively affects the gut microbiota by altering bile acid circulation. After eight weeks of GUDCA administration, blood glucose and lipid levels (total cholesterol and triglycerides) were significantly reduced in mice. Furthermore, the glucose and insulin tolerance test results, insulin levels, and HOMA-IR index of experimental mice were reduced. GUDCA increases the levels of taurine cholic acid and the abundance of *Bacteroides* vulgaris in mice, activating TGR5 and upregulating the expression of UCP-1, which leads to increased thermogenesis in white adipose tissue and induction of intestinal GLP-1 secretion (Chen et al., 2023). Therefore, disturbances in the gut microbiota can reduce the formation of bile acids, hinder the production of free and secondary bile acids (Fang et al., 2022), and weaken the activation of the FXR, resulting in abnormal glucose metabolism. Furthermore, the activation of the TGR5 receptor is weakened, which leads to a decrease in thyroid hormone thermogenesis, GLP-1 levels, and insulin secretion and an increase in blood sugar levels (Massey and Brown, 2021; Xia et al., 2021; Chen et al., 2023; Makki et al., 2023). As a metabolite, the reduction of bile acid (Fang et al., 2022) aggravates this enteric dysbacteriosis and forms a cycle that seriously affects the normal metabolic signaling pathways and leads to the occurrence of DM (Canfora et al., 2022).

### 3.4 Gut microbiota: tryptophan and DM

Tryptophan is an aromatic amino acid and one of the eight essential amino acids found in humans and animals. As a biosynthetic precursor of a large number of microbial and host metabolic pathways, various derivatives can be generated through the kynurenine, indole metabolic, and serotonin pathways, which exert biological effects. The kynurenine pathway is involved in immune activation and inflammatory regulation and is associated with obesity and insulin resistance (Vangipurapu et al., 2020). Tryptophan is catabolized by intestinal microbes and converted into various indole derivatives such as indole acetic acid, indole lactic acid, and indole propionic acid. Tryptophan, kynurenine pathway metabolites, and indole lactate are positively associated with T2DM risk, whereas indole propionate is negatively associated with T2DM risk (Tuomainen et al., 2018). In addition, some compounds produced by tryptophan metabolism are ligands for aromatic receptors (AhRs), which can cause changes in the AhR conformation. Under activation of the AhR pathway reduces GLP-1 and interleukin (IL)-22 production and increases intestinal permeability and LPS translocation, leading to inflammation and insulin resistance. An article published in GUT in 2022, based on an epidemiological cohort, examined prospective associations between circulating levels of host and microbial tryptophan metabolites and the incidence of DM, elucidating the relationship between metabolite-related host genetics, diet, and the gut microbiome (Qi et al., 2022). These results indicated that the transformation of tryptophan metabolism to intestinal microbial indole propionate production through host-microbial interactions could improve the progression of DM.

### 3.5 Gut microbiota: trimethylamine N-oxide and DM

Common symbiotic bacteria produce volatile trimethylamine (TMA), which is oxidized by flavin monooxygenases, primarily FMO3, to trimethylamine N-oxide (TMAO) in the liver. The TMA-FMO3-TMAO pathway is associated with T2DM. The addition of TMAO to the diet increased glucose tolerance in mice fed a high-fat diet. High levels of TMAO bind to and activate PERK, thereby promoting hyperglycemia and metabolic disorders. Previous studies have shown that the circulating levels of TMAO are significantly elevated under diabetic conditions in both human and animal models. Researchers have also found that TMAO is closely associated with adverse events such as diabetes-related retinopathy, coronary heart disease, and kidney disease (Zhang et al., 2021; Yakar et al., 2022; Huang et al., 2023; Yu et al., 2024). Jiang et al. found that TMAO increases vascular permeability and endothelial cell dysfunction under diabetic conditions (Jiang et al., 2024). By analyzing the bone mineral density of 254 patients with T2DM, Zhao et al. found a significant linear correlation between TMAO level and bone mineral density in patients with T2DM, suggesting that increased TMAO levels are associated with osteoporosis and osteoporotic fractures in this patient population (Yuan et al., 2024). Serum creatinine doubles in patients with T2DM and elevated circulating TMAO levels, suggesting that elevated serum TMAO levels are positively associated with the risk of Diabetic Kidney Disease in these patients (Huang et al., 2023; Yu et al., 2024). Therefore, elevated serum levels of trimethylamine oxide may serve as a potential biomarker of diabetes progression.

### 3.6 Brief summary

The preceding discussion introduces the mechanism by which gut microbiota metabolites participate in DM. However, the gut microbiota also influences the incidence of T1DM as an autoimmune disease by altering the immune response. Structural variations in the gut flora damage the integrity of the intestinal barrier, causing increased intestinal permeability and ectopic distribution of certain metabolites throughout the body. This process directly destroys islet β-cells and triggers systemic inflammation and autoimmune processes (Yan et al., 2019; Arora et al., 2021; Zhang et al., 2021; Ng et al., 2022; Fu et al., 2023a; Wu et al., 2023). Furthermore, innate immunity plays a crucial role in the etiology of T1DM. TLRs are important factors in innate immunity and are essential for maintaining intestinal homeostasis. Simon et al. (2020) recently found that compared with NOD TLR4+/+ mice, NOD TLR4-/- mice had an increased risk of diabetes before developing T1DM. In their study, the abundance of *Bacteroides* in the large intestine was higher, whereas that of *Firmicutes* was lower, suggesting that TLR4 expression affected the varieties and quantities of the gut microbiota, influencing the incidence of T1DM. Finally, T cells are significant players in adaptive immune responses, both in fighting pathogens and regulating immune responses to maintain immune homeostasis. Therefore, most studies suggest that enteric dysbacteriosis changes intestinal permeability and the intestinal immune response in the pathogenesis of T1DM (Demirci et al., 2020; Huang et al., 2020; Ma et al., 2020; de Groot et al., 2021; He et al., 2022; Syed, 2022; Wang et al., 2022; Xie et al., 2022; Yuan et al., 2022; Zheng et al., 2022; Diviccaro et al., 2023; Moreira et al., 2023).

In summary, FMT can improve obesity and blood sugar levels in diabetic mice; the mechanism may involve changes in microflora and metabolites, improvement of insulin resistance, and diabetes treatment.

## 4 FMT used to treat DM

### 4.1 Animal experiment

Relatively few studies exist on the treatment of T1DM using FMT. Previous studies have shown that FMT can significantly improve insulin resistance in non-obese diabetic mice. Subsequently, some scholars explored the relevant mechanism and found that the incidence of T1DM in mice in the FMT group was 40.9%, compared with 72.7% in the control group (Zhou et al., 2022). FMT alleviated the islets of NOD mice and slowed the development of T1DM. The mechanism may be related to the remodeling of the intestinal flora of NOD mice, improving intestinal barrier function, affecting the immune response of the gut-pancreas immune axis, concurrently modifying the disorder of amino acid metabolism, and reducing the accumulation of branch-chain amino acids in NOD mice during the islet stage. Researchers are continuing to gain insight into the advantages of FMT in T2DM treatment regimens through animal experiments. Wang et al (Wang et al., 2020). demonstrated that rebuilding the microbiota of T2DM mice using FMT could alleviate hyperglycemia, reverse insulin resistance, and repair damaged islets. For the Kazakh population in China, a healthy human fecal sample was collected after meeting the inclusion criteria for intervention in db/db mice. *Desulfovibrio* and *Clostridium coccoides* levels in the intestines of FMT-treated mice were significantly reduced, whereas *Akkermansia* levels in the feces and histone deacetylase-3 (HDAC3) protein expression in the colon were elevated. Both intestinal target bacteria and HDAC3 correlated with glycolipid levels, and *Myxophilus* levels positively correlated with HDAC3 protein expression (r = +0.620, p = 0.037). Correspondingly, the levels of fasting blood glucose, postprandial blood glucose, total cholesterol, triglycerides, and low-density lipoprotein cholesterol were significantly reduced in FMT-treated db/db mice, whereas the levels of high-density lipoprotein cholesterol were upregulated. Finally, the results of this study suggest that fecal bacteria from KNGT may be used to treat patients with diabetes (Zhang et al., 2020). In contrast, Wang et al. collected stool from Uyghur patients with T2DM to intervene with C57BM/6 mice and found that Uyghur T2DM fecal microbiota transplantation disrupted glucose metabolism by altering the ability of intestinal flora to metabolize BIS and the BAS/GLP-1 pathway (Wang et al., 2022). Zhang et al. transplanted the gut microbiota of healthy or obese T2DM rats into the gastrointestinal tracts of Zucker diabetic fatty (ZDF) and lean Zucker (LZ) rats (Zhang et al., 2020). Using 16S rRNA sequencing and macrometabolomics techniques, diabetic rats transplanted with normal gut microbiota exhibited significant weight loss, lower HbA1c levels, improved glucose tolerance, and enhanced insulin tolerance, compared with the control group. These findings provide evidence that FMT effectively enhances body weight and blood glucose modification in T2DM rats. To explore the impact of FMT on glycometabolism and insulin secretion in mice, a study conducted by Chen et al (Chen et al., 2023). revealed that FMT treatment in db/db mice resulted in significant improvements in various clinical indicators, such as serum insulin levels, fasting blood glucose levels, and oral glucose tolerance, compared with that in the control group. Yang et al (Yang et al., 2023). evaluated the curative effect of FMT, compared with that of metformin (MET) in 28 mice randomly divided into four groups (control, T2DM, MET, and FMT). Fasting blood glucose and low-density lipoprotein levels were reduced in both the MET- and FMT-treated diabetic mice, suggesting that FMT can be used to treat T2DM by improving hyperlipidemia and hyperglycemia. In the study by An et al. (2023), 32 SPF-grade SD rats were used as donors and divided into three groups (control, T2DM, and non-T2DM). Feces were collected, and the supernatant was retrieved after centrifugation. Subsequently, 79 SPF-grade SD rats were divided into a normal saline group (NS group) and an antibiotic group (ABX group). The ABX group was further randomly divided into five groups, and the NS group was divided into two groups according to diet and FMT intervention. In the ABX group, *Ruminococcus gnavus* in the T2DM fecal supernatant group after a high-fat diet was more abundant than that in the transplant control fecal supernatant group and FMT non-intervention group. The levels of blood glucose, serum insulin, total cholesterol, triglycerides, and low-density lipoproteins in rats were higher than those in high-fat diet intervention group. After FMT intervention, the levels of acetic acid and butyric acid were increased, and the expression of GPR41/43 was also significantly increased, indicating that *Ruminococcus gnavus* may increase the risk of T2DM in rats. The gut microbiota, SCFAs-GPR41/43, may play a role in the development of T2DM.

In a nested case-control study, the transfer of different gestational fecal samples from GDM and non-GDM donors to germ-free mice resulted in different patterns of intestinal microbiota colonization and induced hyperglycemia in mice that received GDM donor microbiota (Liu et al., 2020). Similarly, feces from patients with GDM and healthy pregnant women were transplanted into germ-free mice to establish a gestational mouse model, and data from mice at different gestational stages were recorded. The weight and blood glucose levels of the offspring of GDM-FMT mice were higher than those of control offspring (Qin et al., 2022). FMT further suggested that GDM is closely related to abnormal intestinal flora and that the offspring of these women have an increased risk of diabetes. Changes in maternal flora also affect the occurrence of GDM.

FMT was used for the first time in a preclinical model of DN (BTBR ob/ob mice) (Bastos et al., 2022) which revealed that FMT was a safe treatment that prevented weight gain, reduced proteinuria, reduced local expression of TNF-α in the intestine, and potentially improved insulin resistance.

### 4.2 Clinical research

The effect of FMT on the progression of T1DM was investigated by de Groot et al. by assessing the retention of stimulated C-peptide release in a 12-month mixed meal trial (de Groot et al., 2021). The results indicated that pancreatic β-cell function and stimulation levels were retained at 12 months in the FMT group, suggesting that FMT can protect pancreatic β-cell function in patients with diabetes diagnosed 12 months after onset and avoid a drop in endogenous insulin production. Additionally, the study revealed significant alterations in plasma metabolites, specifically MA-GPC (p = 0.02, MWU) and A-GPC (p = 0.02), between the allogeneic FMT group and autogenous FMT group (control group). In another clinical trial, researchers conducted a 1-year therapeutic study involving two teenagers with T1DM who received one to three rounds of FMT and were followed up for up to 30 weeks (He et al., 2022; Zhang et al., 2022). After transplantation, the diabetes-related clinical indicators of both patients showed significant improvement, indicating that FMT was effective and could be maintained to control blood glucose levels in patients with diabetes. FMT notably enhanced insulin resistance in two patients and effectively extended the function of residual β-cells in patients with diabetes receiving FMT. In addition to the therapeutic effects of FMT on T1DM, FMT also ameliorates DM-related complications. A malnourished patient with T1DM who was unable to control blood glucose levels with insulin and experienced severe gastrointestinal symptoms accepted FMT treatment via nasojejunal tube implantation (Xie et al., 2022). No obvious adverse reactions were observed after FMT, and vomiting, nausea, and other symptoms were alleviated. Additionally, body weight and body mass index increased, and trophic status, constipation, and glycemic control (fasting plasma glucose and glycated hemoglobin [HbA1c]) gradually improved. Other clinical indicators, including total protein, albumin, and hemoglobin levels, returned to normal.

In related clinical trials, the health of patients with T2DM improved after FMT. Researchers have found that FMT changes the gut microbiome more quickly than diet alterations, and patients with T2DM show further improvements in blood sugar, blood pressure, lipids, and body mass index. Ding et al. (2022) recruited 20 healthy individuals as baseline controls and 17 patients with T2DM to receive FMT from healthy donors for 12 weeks and evaluated their HbA1c% and metabolic parameter changes. Uric acid, blood sugar, and HbA1c% levels decreased, whereas postprandial C-peptide levels increased significantly after 12 weeks. Therefore, researchers believe that FMT can ameliorate insulin function and glycometabolism in T2DM and that some patients may benefit from FMT. In a randomized trial (Ng et al., 2022), 61 participants with T2DM were divided into three groups for 24 weeks of FMT and lifestyle intervention; combining the two methods led to more favorable variations in the participants’ microbiomes and improved lipid and liver stiffness. Wu et al (Wu et al., 2023). compared the effect of FMT alone, MET alone, and FMT plus MET on patients with T2DM and their gut microbiota. In their study, 31 newly diagnosed patients with T2DM were randomized to receive MET, FMT, or FMT plus MET and were followed up from week four, with blood and stool samples collected periodically. Compared with those of MET alone, blood glucose levels, HbA1c levels, and insulin resistance were clearly decreased after FMT treatment. Additionally, uric acid and triglyceride levels were significantly decreased, and fasting blood sugar and HbA1c levels were decreased when FMT was combined with MET treatment.

Cai et al. (2018) performed FMT in a patient with DPN combined with hypertension, poor blood sugar control, and anxiety caused by long-term pain. After the first FMT, the patient’s pain was alleviated; blood sugar levels, blood pressure, and lipid levels decreased significantly; and weight loss was observed. Although nutritional neuropathy and painkillers are the main treatment methods for diabetic neuropathy, the patient did not use any analgesic drugs after FMT. Furthermore, the patient’s HbA1c, blood sugar, and uric acid levels decreased significantly, while C-peptide levels significantly increased after meals. Therefore, the fecal microbiota provided by healthy donors was crucial for improving glycometabolism in T2DM. FMT also improved insulin resistance and mitigated islet dysfunction. In a randomized, double-blind trial of diabetic distal symmetrical polyneuropathy (DSPN), 22 patients with DSPN who received fecal microbiota transplants from healthy individuals showed significant improvements in symptoms, independent of blood glucose control, compared with 10 patients who received placebos (Yang et al., 2023).

These experiments suggest that re-establishing intestinal microecological balance through FMT may be an alternative remedy for DM and its associated diseases (**Tables 2**, **3**)

**Table 2:** Animals experiments of FMT for treatment of DM.

Donors	Animals	FMT routes	FMT-effects on DM	FMT-effect on gut microbiota	Ref.
NOD mice, C57BL/6 mice	NOD mice	Oral gavage	1. Alleviates islet inflammation and slows the occurrence of T1DM in NOD mice 2. Correcting the disorder of amino acid metabolism and reducing the accumulation of branched-chain amino acids in NOD mice during islet stage	1. Remodeled intestinal flora and improved gut microbiota function in NOD mice 2. Influenced the immune response of entero-pancreatic immune axis	(Zhou et al., 2022)
Kunming (KM) mice	Kunming (KM) mice	Oral gavage	1. Reduced FBG and improved glucose tolerance 2. Insulin resistance and pancreatic islet β-cells were improved 3. Inflammatory response decreased, β-cell apoptosis inhibited	Restore the balance of gut microflora	(Wang et al., 2020)
Kazaks with normal glucose tolerance (KNGT)	db/db mice	Oral gavage	1. Fasting blood glucose, postprandial glucose, total cholesterol, triglyceride, and low-density lipoprotein-cholesterol were significantly downregulated 2. High-density lipoprotein-cholesterol levels were upregulated	1. *Desulfovibrio* and *Clostridium coccoides* levels in gut were significantly decreased 2. Fecal levels of *Akkermansia muciniphila* and HDAC3 protein expression were increased	(Zhang et al., 2020)
Uygur T2DM	C57BL/6 mice	Oral gavage	1. Insulin and oral glucose tolerance are impaired 2. Deoxycholic acid increased and tauro-β-muricholic acid and the non-12-OH BA:12-OH BA ratio decreased in plasma	The ability of intestinal flora to produce *deoxycholic acid* improved	(Wang et al., 2022)
Healthy, obese-T2DM	ZDF and LZ rats	Oral gavage	1. Altered the glycolipid metabolism phenotype in ZDF rats	1. ZDF rats: *Bacteroides* showed high expression, *Roseburia, Coprococcus, Rothia*, and *Allobacum* decreased 2. Trend in the group of transplanted with LZ microbiota was opposite	(Zhang et al., 2020)
db/m mice	db/db mice	Oral gavage	A series of clinical indicators were relieved, including fasting plasma glucose, serum insulin and oral glucose tolerance test among others	1. db/db+PBS mice: *Ruminococaceae, Porphyromonadaceae* decreased, *Rikenellaceae* and *Lactobacillaceae* increased 2. db/db+FMT groups are opposite	(Chen et al., 2023)
C57BL/6J mice	C57BL/6J mice	Oral gavage	FMT had a curative effect on T2D by ameliorating hyperlipidemia and hyperglycemia	1. At the phylum level, the abundance of *Bacteroidetes* increased after FMT and MET treatments compared with the T2D group. 2. At the genus level, the FMT group and MET group down-regulated harmful bacteria such as *Lachnospiraceae_Clostridium, Helicobacter*, and *Erysipelotrichaceae_Clostridium* compared to the T2D group. 3. FMT could restore the disorders of gastrointestinal microbiota in T2D mice	(Yang et al., 2023)
Control group (fed with an ordinary diet) HFD group (fed with a high-fat diet)	SPF-grade SD rats	Oral gavage	T2DM-susceptible flora transplantation could increase the level of blood glucose, decrease the level of serum insulin, and promote IR, slowing down lipid metabolism in rats.	1. *G_Ruminococcus_gnavus_*group might make rats more susceptible to T2DM 2. T2DM-susceptible flora transplantation increased the susceptibility to T2DM in rats 3. GM -SCFAs-GPR41/43 may play a role in the development of T2DM.	(An et al., 2023)
GDM and non-GDM	C57/BL6 GF mice	Oral gavage	Hyperglycemia was induced in mice that received GDM donor microbiota	1. The relative abundance of *Akkermansia* was negatively associated with blood glucose levels 2. The relative abundance of *Faecalibacterium* was positively related to inflammatory factor concentrations.	(Liu et al., 2020)
GDM: three individuals Non-GDM: three individuals	C57/BL6 GF mice	Oral gavage	1. An elevated blood glucose 2. An inflammatory factor expression (TNF-α, CXCL-15, and IL-6), 3. A hepatic fat deposition. 4. The offspring of GDM-FMT mice had higher body weight and blood glucose levels than the control offspring	1. GDM-FMT group: a lower relative abundance of *Akkermansia* and *Faecalibacterium* 2. The offspring of GDM-FMT mice: a lower relative abundance of *Akkermansia* and *Parvibacter*; a higher relative abundance of bacteria such as *Oscillibacter*, *Romboutsia*, and *Harryflintia*	(Qin et al., 2022)
BTBR wild-type	BTBRob/ob mice	Rectal route	1. Prevented body weight gain 2. Ameliorated insulin resistance	1. Reduced albuminuria and tumor necrosis factor-α (TNF-α) levels within the ileum and ascending colon 2. FMT was associated with the abundance of the *succinate-consuming Odoribacteraceae bacteria* family throughout the intestine	(Bastos et al., 2022)
NG, DSPN, non-DSPN	db/db mice	Oral gavage	Aggravation of peripheral neuropathy	M-DSPN group: the structure of GM is significantly, poor intestinal barrier function, LBP increased	(Yang et al., 2023)

**Table 3:** Clinical studies of FMT for the treatment of DM.

Diseases	Countries	Sample	Pathways	Treatment	Potential mechanisms	Efficacy and safety	Ref.	Prognosis
T1DM	Netherlands	10	Nasoduodenal tube	Other: encapsulated autologous FMT	Stabilization of the β-cell destruction and extending or even bringing back the honeymoon period	FMT halts the decline in endogenous insulin production. Safe	(de Groot et al., 2021)	No serious clinical AEs nor adverse changes in plasma biochemistry
T1DM	China	1	Nasojejunal tube implantation	FMT	Specific bacteria involved in several metabolic pathways	Constipation, nutritional status, and blood glucose control (fasting blood glucose, HbA1c) gradually improved Safe	(Xie et al., 2022)	No adverse reactions during the FMT treatment
T1DM	China	2	Oral liquid capsules(most)	FMT	Increase in *B. cellulosilyticus*; increased in abundance of *A. shahil*	FMT could control the FBG level; the mean glycated hemoglobin was significantly better controlled. Safe	(He et al., 2022; Zhang et al., 2022)	Two patients had adverse no effects after FMT
T2DM	China	17	Transendoscopic enteral tubing	Largely determined by the baseline gut microbiota composition.	Restoration of a healthy gut microbiota could lead to a potential clinical benefit	Certain T2DM patients can potentially benefit from FMT Safe	(Ding et al., 2022)	No significant AEs or gastrointestinal symptoms were found to be related to the FMT treatment
T2DM	China	61	Solution was injected into the distal duodenum via OGD under conscious sedation.	FMT, lifestyle modification	Enhancing microbiota engraftment is a prerequisite for FMT to be a potentially useful treatment for metabolic syndrome.	Repeated FMTs enhance the level and duration of microbiota engraftment in obese patients with T2DM Combining lifestyle intervention with FMT led to more favorable changes in recipients’ microbiota and improvement in lipid profile and liver stiffness Safe	(Ng et al., 2022)	None of the AEs were related to intervention. There was no mortality.
T2DM	China	29	Solution was injected via the nasointestinal tube to the anterior jejunum.	FMT, metformin	FMT with or without metformin significantly improve insulin resistance and body mass index and gut microbial communities of T2DM patients by colonization of donor-derived microbiota.	Fasting and postprandial blood glucose, HbA1c, and insulin resistance decreased significantly without causing hypoglycemia or dyslipidemia after FMT treatment Safe	(Wu et al., 2023)	T2DM patients displayed no adverse reactions after FMT
DPN	China	1	The fresh fecal microbial suspension was delivered to terminal ileum through endoscopy under anesthesia	FMT	/	Glycemic control was improved, with a remarkable relief of the symptoms of painful DN in particular Safe	(Cai et al., 2018)	No obvious adverse effects were observed during the FMTs and follow-up observation-testing
DSPN	China	32	/	FMT	The key bacteria may mediate the protective effect of the gut microbiota The mediating role of the two competing guilds in successful FMT (guild 1, guild 2)	FMT alleviated the severity of peripheral neuropathy in patients with DSPN FMT improved gut-barrier integrity and systemic inflammatory status in patients with DSPN Safe	(Yang et al., 2023)	No serious adverse events were documented during the trial

AE, adverse event; DN, diabetic nephropathy; FBG, fasting blood glucose; FMT, fecal bacterial transplantation; HbA1c, glycated hemoglobin; T2DM, type 2 diabetes mellitus

"/" indicates that there are no specific details or relevant content in the literature.

### 4.3 Role of gut microbiota in the therapeutic approach for treating diabetes

**Figure 5:**
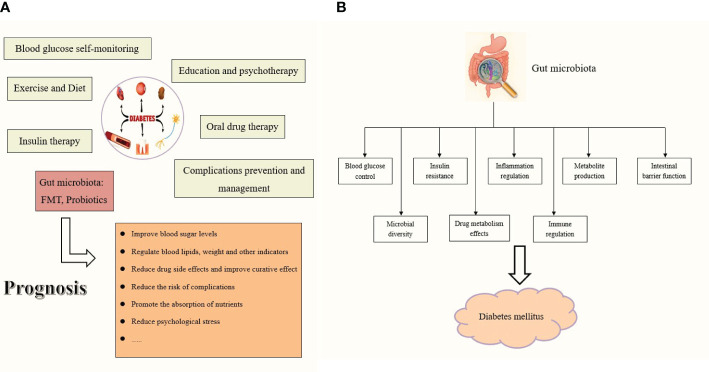
**(A)** The role of gut microbiota in DM prognosis. **(B)** The role of gut microbiota as a therapeutic to treat the diabetes mellitus.

The gut microbiota can be broadly classified into three categories: beneficial, harmful, and neutral bacteria (Gilbert et al., 2018; Adak and Khan, 2019; Zhou et al., 2020; Aggarwal et al., 2023; Fu et al., 2023b; Wang et al., 2023). Beneficial bacteria, also known as probiotics, primarily include various *Bifidobacterium* and *lactobacillus*, which synthesize various vitamins, participate in food digestion, promote intestinal peristalsis, inhibit the growth of pathogenic bacteria, and decompose harmful toxic substances. During the long-term evolutionary process of the gut microbiota, different types of flora; the flora and host; and the flora, host, and environment have always maintained a dynamic balance. Therefore, when the floral structure is relatively stable, it does not cause disease in the host. Existing literature shows that gut microbiota diversity in patients with diabetes is reduced, and the ratio of *firmicutes* to *bacteroides* is significantly reduced (Schloissnig et al., 2013; Zhang et al., 2021; Del Chierico et al., 2022; de Vos et al., 2022; Guo et al., 2022; Zhou et al., 2022; Crudele et al., 2023; Wu et al., 2023). Human health is closely related to the structure of probiotics in the gut, such as *bifidobacterium*, which is negatively correlated with blood sugar, blood pressure, lipids, and body mass index. Specifically, *Bifidobacteria*, *Bacteroides*, and *Coprologis* are negatively correlated with DM, whereas *Ruminococcus*, *Fusobacterium*, and Cyanobacteria are positively correlated. Diabetic microangiopathosis is positively correlated with *Bacteroides* abundance and negatively correlated with *Prevotella* abundance (Hong et al., 2022; Li et al., 2023; Sapra and Bhandari, 2023; Jia et al., 2024; Kanbour et al., 2024; Ma et al., 2024). *Pasteurella bacteria* can be used as a microbial feature to distinguish DR from DM, and *Blautia* may be a protective factor against DN. In addition, the gut microbiota plays a crucial role in the human metabolic system, and various metabolites are produced by the gut microbiota in different ways. As research deepens, some metabolites may not only participate in the pathogenesis of DM, but may also serve as important potential biomarkers indicating the complications of diabetes. The increase in LPS from gram-negative bacteria, SCFAs, secondary bile acids, trimethylamine, indole, tryptophan, and other gut microbiota derivatives aggravates inflammation and metabolic dysfunction and promotes the onset and progression of diabetes. The progression of diabetes also leads to gut microbiota disorders, manifested as the reduction of probiotics, increase of harmful bacteria, and introduction of disease caused by neutral bacteria; the interaction between these three aggravates disease. Generally, intestinal flora, especially probiotics, can improve the absorption of sugars in the intestine, regulate blood sugar, inhibit inflammation, protect islet cells, maintain the integrity of the intestinal barrier, reduce the entry of harmful substances into the blood, and reduce systemic inflammatory responses in patients. They can regulate the host metabolic pathway, improve insulin resistance, increase insulin sensitivity, affect intestinal pH and enzyme activity, promote the absorption and utilization of hypoglycemic drugs, promote nutrient absorption, and participate in lipid metabolism, thereby controlling blood sugar levels, reducing the risk of complications, and improving the prognosis and quality of life of patients with diabetes (**Figures 5A, B**).

While the mainstream treatment for diabetes currently relies on insulin replacement and hypoglycemic drugs, a paradigm unlikely to change in the immediate or distant future, the application of FMT introduces new perspectives and directions for the treatment of DM. Dietary habits, lifestyle modifications, pharmaceuticals, and supplementation with exogenous probiotics can all regulate gut microbiota, with FMT being the most direct and effective intervention. Initially, FMT served as a tool to isolate probiotics and enhance the gut microbiota ecology in diabetes. Subsequently, in animal experiments, FMT directly augmented the diversity of the gut microbiota, restored intestinal function, improved the *in vivo* metabolic status, delayed the progression of diabetes, and exerted a positive feedback effect. Regarding current symptomatic treatment of diabetes, FMT is anticipated to address the root cause of diabetes by adjusting the intestinal microecological balance and improving insulin resistance, implicating multiple mechanisms such as inflammation and metabolism. By examining the changes in metabolites before and after FMT, we can gain a deeper understanding of the role of potential markers and further elucidate the pathogenesis of gut microbiota in DM. Finally, the safety and feasibility of FMT were verified using animal models. Considering drug allergies, intolerance, and poor response to traditional treatment in some patients, FMT was administered one to three times. Subsequently, clinical indicators such as blood glucose level, hemoglobin level, BMI, and islet function showed significant improvement, thereby affirming that FMT is a viable option. Additionally, the long-term effects of FMT may be more stable and have fewer adverse effects than those of traditional drug therapy, which may necessitate constant dose adjustments or drug changes.

## 5 Clinical safety

No obvious adverse events have been reported in recent studies regarding the use of FMT for DM. The majority of diabetic patients do not experience adverse reactions following FMT treatment, which appears to improve HbA1c and blood glucose levels and islet function, and does not induce hypoglycemia or hypolipidemia. One study (Zhang et al., 2020) indicated that the initial transplantation of gut microbiota caused a temporary inflammatory response in patients. However, once patients adapted to the transplanted microbiota, the inflammatory markers returned to normal levels. Concurrently, no significant abnormalities were observed in certain indices before and after treatment, further suggesting that FMT did not inflict damage to the kidneys, liver, or other organs. When a patient with diabetes and gastrointestinal symptoms (Xie et al., 2022) experienced recurrent nausea and vomiting, existing drug treatments were effective. After FMT, the patient’s gastrointestinal discomfort decreased.

## 6 Conclusion

Previous studies have underscored the therapeutic potential of FMT for DM; however, several issues remain unresolved. Currently, large-sample randomized controlled trials investigating FMT for the treatment of DM are insufficient. Therefore, further research is required to ascertain long-term efficacy and safety for this treatment. Additionally, the mechanisms underlying the effects of FMT on DM and their broader impact on DM are discussed.

As a direct and potent intervention for gut microbiota disorders, FMT holds promise in predicting the clinical treatment of DM. It can restructure the gut microbiota in patients with diabetes, restore the diversity of the original gut microbiota, inhibit inflammatory responses, and modulate immune responses. FMT can also stabilize the metabolic state of the body. Clinical indicators related to DM, including blood glucose, insulin, C-peptide, and HbA1c levels, also improved. Although research on FMT in relation to GDM and other specific types of diabetes is limited, significant progress has been made. Ongoing studies aim to further explore these complications. FMT not only regulates the metabolic levels of diabetic patients but also treats other DM-related diseases, such as obesity, metabolic syndrome, and neuropsychiatric diseases, thereby achieving comprehensive intervention and management for these patients.

FMT represents a novel concept and technology for treating gut microbiota-related diseases. Despite these limitations, FMT remains a crucial tool for investigating the role of microorganisms in the pathogenesis of chronic diseases. As a new form of individualized microecological treatment, it can regulate blood glucose levels and metabolic status; reduce patient dependence on drugs and their side effects; improve patient prognosis and quality of life; and provide a more effective, safer, and longer-lasting treatment program.

Research has indicated a correlation between individual flora, blood sugar, and other indicators. Therefore, the feasibility of single-flora transplantation therapy is worth exploring. Currently, significant challenges inhibit the clinical promotion of FMT for the treatment of DM. The selection of FMT donors, transplantation mode, dose frequency, patient acceptance, and need for dietary intervention after FMT should be further studied. Overall, based on existing studies, DM treatment has been proven to be effective and feasible, and we believe that standard, high-quality, and safe FMT will provide hope to patients with DM.

## Author contributions

JZ: Writing – original draft. HW: Writing – review & editing. YL: Writing – review & editing. MS: Writing – review & editing. MZ: Writing – review & editing. HZ: Writing – review & editing. JC: Writing – review & editing.
